# A New Technology for Applanation Free Corneal Trephination: The Picosecond Infrared Laser (PIRL)

**DOI:** 10.1371/journal.pone.0120944

**Published:** 2015-03-17

**Authors:** Stephan J. Linke, Andreas Frings, Ling Ren, Amadeus Gomolka, Udo Schumacher, Rudolph Reimer, Nils-Owe Hansen, Nathan Jowett, Gisbert Richard, R. J. Dwayne Miller

**Affiliations:** 1 Department of Ophthalmology, University Medical Center Hamburg-Eppendorf (UKE), Martinistrasse 52, 20246, Hamburg, Germany; 2 CareVision/Clinica Baviera Germany and Austria, Martinistrasse 52, 20246, Hamburg, Germany; 3 Max Planck Institute for the Structure and Dynamics of Matter, Luruper Chaussee 149, 22761, Hamburg, Germany; 4 Department of Anatomy and Experimental Morphology, University Medical Center Hamburg-Eppendorf (UKE), Martinistrasse 52, 20246, Hamburg, Germany; 5 Heinrich Pette Institute, Leibniz Institute for Experimental Virology, University Medical Center Hamburg-Eppendorf (UKE), Martinistrasse 52, 20246, Hamburg, Germany; 6 Department of Otorhinolaryngology, University Medical Center Hamburg-Eppendorf (UKE), Martinistrasse 52, 20246, Hamburg, Germany; University of Missouri-Columbia, UNITED STATES

## Abstract

The impact of using a Femtosecond laser on final functional results of penetrating keratoplasty is low. The corneal incisions presented here result from laser ablations with ultrafast desorption by impulsive vibrational excitation (DIVE). The results of the current study are based on the first proof-of-principle experiments using a mobile, newly introduced picosecond infrared laser system, and indicate that wavelengths in the mid-infrared range centered at 3 μm are efficient for obtaining applanation-free deep cuts on porcine corneas.

## Introduction

More than a century after the first successful corneal transplant was performed by Eduard Zirm in 1905, numerous advances in surgical techniques and medical therapies have dramatically improved the prognosis and success of corneal grafting [1]. The widespread use of refractive laser eye surgery and the tremendous technological advance with development and incorporation of corneal lamellar procedures have led to the emergence of new transplant techniques and the use of the laser platform in corneal transplant surgery. First attempts to incorporate laser technology into the field of corneal transplants were described by Naumann et al. [2]. The introduction of the femtosecond (FS) laser technology in corneal surgery was initially proposed in the late 1990s [3]. However, astigmatism after penetrating keratoplasty is currently one of the major hurdles that corneal surgeons have to deal with [1].

Postoperative outcomes for FS keratoplasty had significant improvement in astigmatism before the 6 month postoperative follow-up and earlier suture removal was possible [4]. However, although limited by their methodology in some points, recent reviews [4–6] comparing FS versus manual mechanically guided penetrating keratoplasty have also shown no final differences in terms of astigmatism and visual outcome. Thus, at present, arguments could be raised against using FS laser keratoplasty [4–6].

Better docking systems with liquid interfaces that do not distort corneal geometry or even contact-free laser cutting might be options to solve the current problems. Improvements in reducing the laser-pulse collateral tissue damage would help in decreasing the endothelial cell damage and the preservation of collagen fibers. The second main drawback of FS laser-assisted corneal trephination is the limited capability for cutting scarred tissue. For surgery on healthy cornea, the usual wavelengths of 800 nm and 1 μm can be used, while edematous and scarred cornea require specific optimization as suggested by Crotti et al.[7,8].

The corneal incisions presented here result from laser ablations with ultrafast desorption by impulsive vibrational excitation (DIVE) [9,10]. The DIVE principle is embodied in a picosecond infrared laser (PIRL). The output wavelength of 3 μm delivered by the PIRL is specifically tuned to the vibrational absorption band of the water molecule, where the infrared radiation is strongly absorbed upon incidence within a few microns [11].

The aim of the present study was to gather proof of principle data on a new PIRL (3 μm) and its ability to cut corneal tissue.

## Methods

### Materials

Freshly enucleated porcine globes were obtained from a local slaughter on Monday mornings between 5 and 7am (Radbruch Fleischerei GmbH, Hamburg, Germany), kept refrigerated, and irrigated using a physiological solution. The samples were delivered to our department at 10am and the experiments took place during the afternoon of the same day. Porcine corneoscleral discs were excised from the pig eyes by a trained preparator using a hand trepan. To investigate the repeatability of ablation characteristics and reliability of laser settings, we used eight different ablation times (1–8 s). For each time group, five ablations were performed on separate porcine corneas using the same laser settings. Before ablation experiments were performed, the corneal epithelium was removed mechanically by scraping with a plastic spatula. For the laser process, all probes were placed on an artificial anterior chamber, and brought into position using the focusing facility of the PIRL. All ablation experiments and characterization measurements were performed within 10 h after excision. In total, 40 porcine cornea were used.

### Laser System

The ultimate application goal of the laser under development is, in its first stage, applanation-free cutting of corneal tissue. Therefore, certain requirements are laid on the laser beam-scanning system, which must provide computer-controlled formation of a circular trephination of varying diameter on the curved corneal surface. The curvature of the cornea has to be taken into consideration, i.e. cutting should be centered on the corneal vertex.

**Fig 1:**
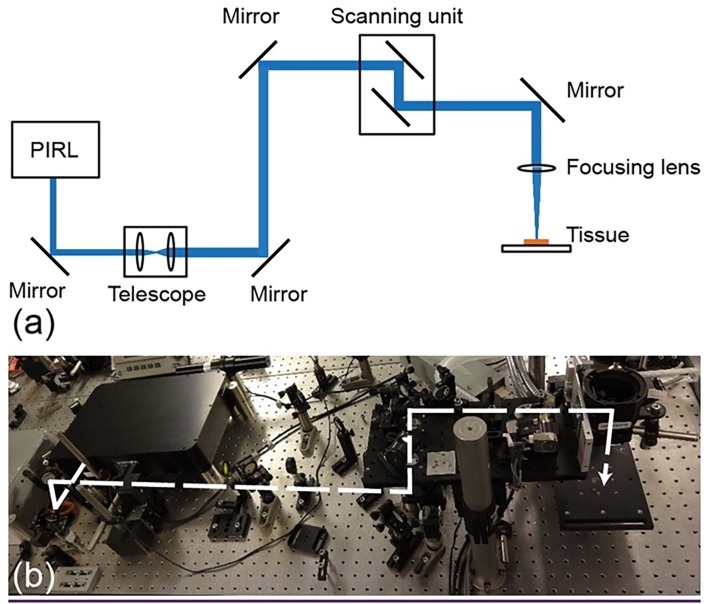
(a) Sketch and (b) experimental set-up for ex vivo non-contact PIRL circular trephination. The dashed line indicates the path of the PIRL beam.

The driving laser system used here was a PIRL-HP2-1064 OPA-3000 (Attodyne Inc., Canada). The output from the PIRL is specified for a wavelength of 3000 ± 90 nm, a pulse duration of 300 ps and a repetition rate of 1 kHz (the output power of 0.70 W). The PIRL beam was delivered to the sample by a homemade system, which also comprised a scanning mechanism (Fig. 1). A distinct ablation pattern, i.e. circle, line, rectangle or disk, can be chosen, and its dimensions defined by the user. In our study, circular 4-mm-diameter ablation patterns were employed for the incisions, and the PIRL beam was scanned accordingly to produce such patterns. The current PIRL output beam must be focused to a tight spot to achieve a significant photon density for ablation to occur; however, the prototype does not possess any autofocus function. The main beam and pulse characteristics of the laser output are summarized in Table 1. For all circular cuts, the PIRL was operated with scan speed set to 120mm/s and repetition rate of 15 Hz.

**Table 1:** Summary of the main beam and pulse characteristics of the laser output compared to currently available laser systems in corneal refractive surgery.

	Excimer	FSL Typ1^1^	Femtosecondlaser Typ2^2^	Near infrared^3^	Mid infrared (Picosecond InfraRed Laser)^4^
**Parameter**					
Wavelength	193 nm	1045 nm	1045 nm	1650 nm	3000 nm
Pulse repetition rate	> 200 Hz	> 60 kHz	10 MHz	10 kHz	1 kHz
Pulse width	20–200 ns	200–350 fs	250 fs	600 fs	300 ps
Output optical power	1.6 mJ	3 μJ	60 nJ	2–20 μJ	460 μJ
Transverse beam diameter	950 μm	3–4 μm	1–2 μm	1 μm	124 μm
Applanation	no	yes	yes	yes	no

Manufacturer:

1: AMO/ Intralase FS, Alcon/ Wavelight FS200, Bausch + Lomb/ Victus, Zeiss Meditec/ VisuMax;

2: Ziemer-LDV 6;

3+4: Prototype

### Histology

Immediately after ablation, tissue samples were fixed in phosphate-buffered 3.5% formaldehyde. Specimens were then embedded in paraffin, cut into 4-um thick sections, and stained with hematoxylin and eosin (H.E., Merck, Darmstadt, Germany). Stained samples were then scanned using MIRAX SCAN (Carl Zeiss Microimaging GmbH, Jena, Germany). Width measurements and histological examinations were carried out using Aperio ImageScope software (Aperio Technologies, Inc., Vista, CA)

The darkly colored edges in the region of the cut were measured; in each sample, the spot size with largest diameter was used to calculate a mean spot size of each ablation group. To rule out that the histological results could stem from elastic fibers, additional samples were stained with resorcin fuchsin dye.

### Environmental Scanning Electron Microscopy (ESEM)

The tissue samples were contrasted with 1% osmium tetroxide in PBS (phosphate-buffered saline) for 30 min and then washed thoroughly. The hydrated tissue blocks were analyzed in backscattered electron (BSE) mode using an XL30 environmental scanning microscope (FEI, Hillsboro, Oregon, USA), equipped with a Point Electronic (Halle, Germany) DISS5 digital image scanning system (3.5 Torr).

### Infrared thermography

A PIR uc 180 thermo camera (InfraTec, Dresden, Germany) with a spectral range of 7.5–13 mm was used to avoid capturing the thermal signal of the 2.94-mm beam. Real-time thermal images (at 100 frames/s) were captured for all ablation trials. The maximal temperature within the ablation path, the temperature at points 1 and 2 mm away from the ablation path and the baseline temperature distant from the irradiated area were measured. Data were analyzed using IRBIS 3Plus software (InfraTec).

## Results

**Fig 2:**
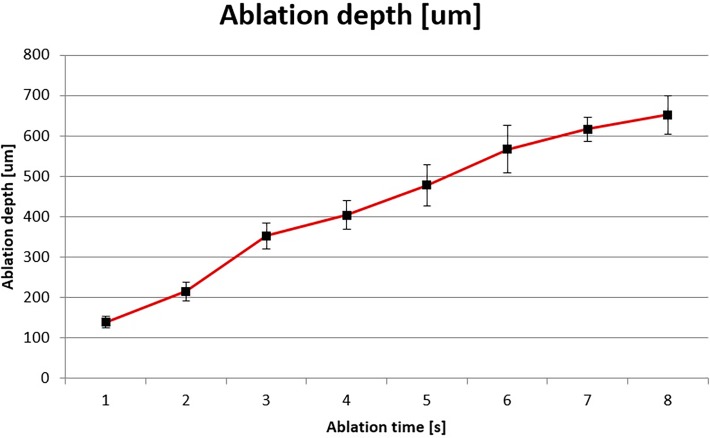
Average depth of ablation with time (compare data in Table 2).

Within each ablation group, relatively stable reproducible ablation depths were obtained (Table 2, Fig. 2). The standard deviation of mean ablation depth was approximately 10% of the ablation depth in each group, respectively. This was more pronounced in ablation groups 1 to 6 sec. The increase in the depth of the corneal ablation was approximately proportional to duration of the ablation. After 7–8 s of ablation, a plateau in depth was reached.

**Table 2:** Summary of the average depth of ablation with time.

Duration of ablation [s]	Average depth of ablation [μm]	Average change between ablation steps* [μm]	Standard deviation [μm]
1	139		±15
2	215	76	±24
3	353	138	±32
4	404	51	±36
5	478	74	±51
6	567	89	±59
7	617	50	±30
8	652	45	±48

*mean increase in ablation depth [um] with increasing ablation time [sec].

**Fig 3:**
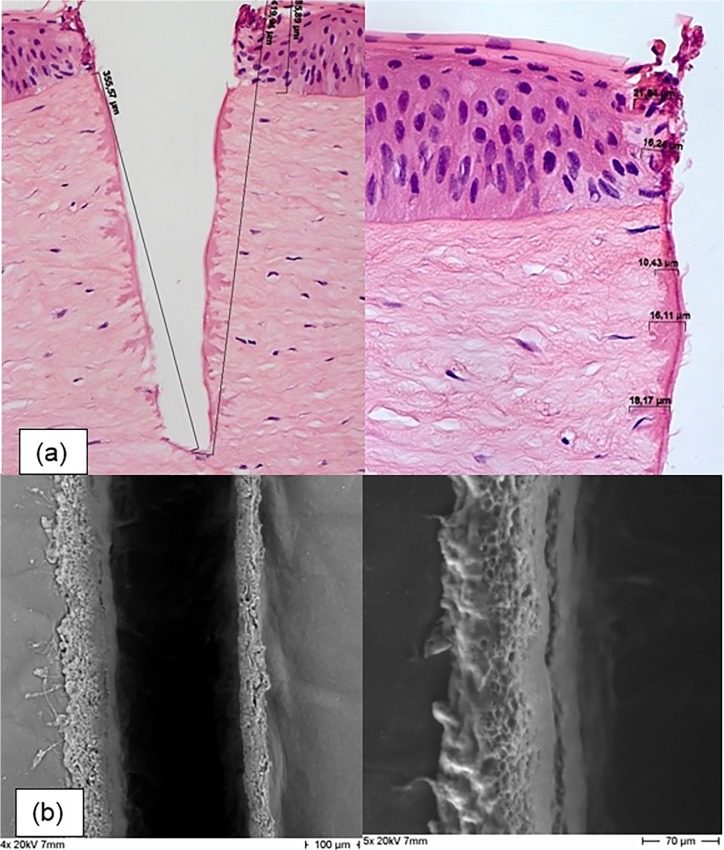
(a) histological, (H.E. staining, magnification 200x and 400x), and (b) ESEM view of circular 4-mm PIRL incisions with 4 sec ablation time in healthy corneal tissue. All methods show precise cutting edges of the PIRL incision deep into the corneal stroma. The H.E. stain (a) reveals minor evidence of thermal effects on maximum the first layer of cells adjacent to the PIRL incision at the PIRL-tissue interface.

**Fig 4:**
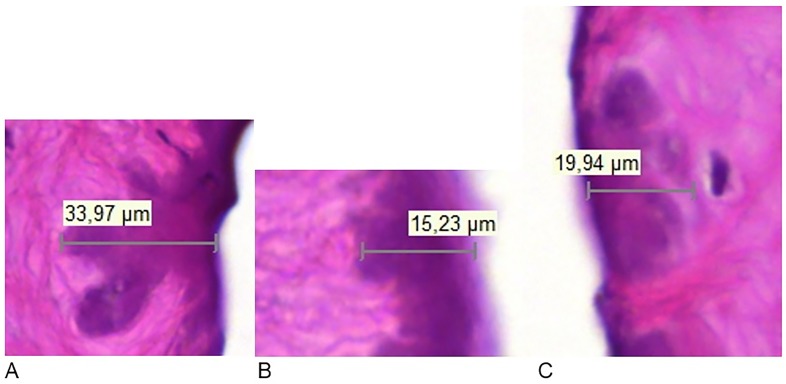
Examples of thermal effect spots of three different ablations times (A: 1sec; B: 4sec; C:8sec).

**Fig 5:**
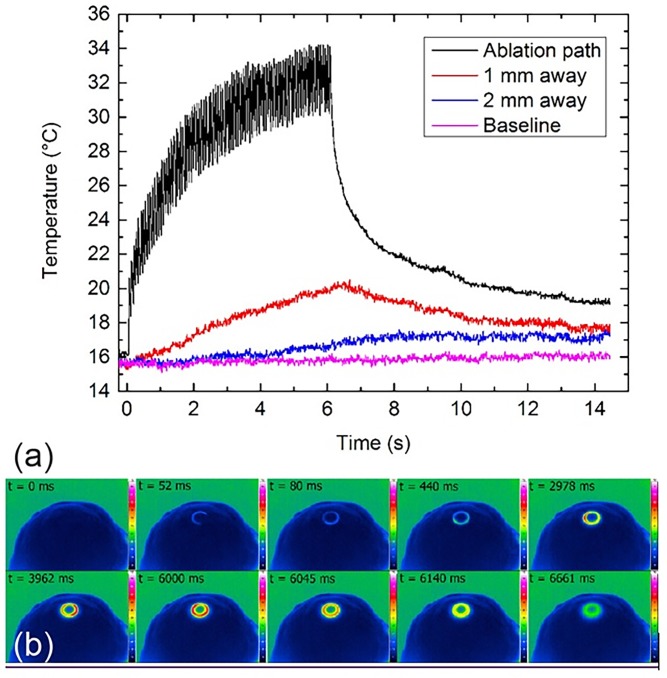
Thermal camera: (a) temperature curves during the 6-s ablation measured at the ablation path, 1 mm and 2 mm away from the ablation path and at the baseline; (b) sequence of thermographs taken from the top view of a porcine cornea placed on the artificial chamber during the PIRL ablation. The ablation path was a circular pattern. After 52 ms, the footprint of temperature increase induced by the PIRL ablation can be easily recognized on the porcine cornea. The temperature is presented in color scale ranging from dark blue (16°C), green (22°C, ambient air), yellow (27°C), red (32°C) to white (37°C, not reached in the experiment).

No evidence of elastic fibers was found. Therefore, the more darkly stained areas at the edges of the ablation cuts were classified as thermal effects on maximum the first layer of cells adjacent to the PIRL incision (Figs. 3 and 4). The width of these areas varied in extent and was independent of ablation time and temperature change (Table 3).The data from the thermal camera showed that ablation induced an increase in temperature in the area of ablation, which increased with the length of ablation. Ablation for 6 s resulted in an increase in temperature in the tissue to a maximum of 34°C; an increase of 18°C (Fig. 5).

**Table 3:** Spot size and standard deviation [μm] calculated as mean value from the spot sizes with largest diameter of samples of three different ablations times (A: 1sec; B: 4sec; C: 8sec).

Ablation time [s]	Spot size and Standard Deviation [μm]	Range (min/max) [μm]
1	18.16 (±13.08)	8.51 to 33.97
4	20.87 (±4.53)	15.23 to 24.76
8	25.57 (±4.73)	19.94 to 31.00

## Discussion

Unlike LASIK, keratoplasty needs lasers that are operational in the volume of scattering tissue [12]. Clinical experience confirms that cutting scarred tissue, e.g. opacified herpetic cornea, cannot be performed with modern FS-assisted platforms in a non-contact procedure [7]. Corneal treatment with photoablation in the mid-infrared range was proposed 20 years ago [13,14]. The Vanderbilt free-electron-laser (FEL) produces 5-μs macropulses at 10 Hz, with each macropulse consisting of 1-ps micropulses at 3 GHz. [15] Experimentally, FEL can ablate soft biological tissues with high efficiency and remarkably little collateral damage. However, FEL has never been used clinically as until now they have comprised large immobile units that are not suitable for patient treatment.

The results of the current study are based on the first proof of principle experiments using a mobile, newly introduced PIRL system, and indicate that wavelengths in the mid-infrared range centered at 3 μm are efficient for obtaining applanation-free deep cuts on porcine cornea.

The key design concept of these experiments is the use of infrared pulses, tuned to one of the dominant vibrational states of the tissue, with pulse durations that are short enough to deposit heat through ultrafast vibrational relaxation but with intensities small enough to avoid plasma formation [9, 16]. The pulse width of the PIRL resides in the range of ten to a few hundred picoseconds, which represents a time window between complete thermalization of individual vibrational excitation [17] and thermoacoustic relaxation time of the excited volume [9]. There is an abundance of water in tissue (e.g. cornea), and the water molecule is selectively excited by the PIRL as a propellant to ablate biological complexes directly into the gas phase [10] in an intact manner, without thermal or shockwave damage to the lateral tissue [18]. The strong absorption and the particular pulse width ensure the confinement and the most efficient coupling of optical energy to phase transition of the tissue [9], which leads to the ablation of the tissue. Therefore, for ablation, the PIRL represents a novel laser scalpel that could theoretically operate at the precision of single cell width with minimal to no scar formation [18].

Compared with FS lasers on the market for medical applications, the PIRL delivers a slightly longer pulse width to prevent breaking weaker bonds or causing undesirable perturbations of molecules [9]. The pulse repetition rate and the optical fluence of the PIRL are lower than those of FS lasers, including those operating at low energy photodisruptive process. An essential difference from the photodisruptive process is that the DIVE process realized by the PIRL does not induce plasma formation or cavitation [9].

Furthermore, applanation during femtotrephination for keratoplasty may result in an oval-shaped recipient bed, which limits the theoretical perfect congruency of donor and recipient [19–21]. Although some studies have shown improved refractive outcome after FS-laser-assisted keratoplasty (fKP), other studies have not been able to confirm reduction of postoperative astigmatism compared to mechanical trephination [5, 19–21]. In summary, so far fKP has not been able to fulfil unequivocally the high expectations and hopes placed in it.

The nature of the residual tissue damage generally depends on the parameters of the laser beam, the type of laser-tissue interactions, and the tissue properties [15, 22]. The most important laser parameters in this regard are wavelength, pulse duration, and pulse fluence, which is defined as the pulse energy per unit area. In a tissue, the optical absorption length, *dABS*, is dependent on wavelength. If the absorption length is not much longer than the wavelength, light scattering can be neglected. To minimize collateral damage, it is essential to minimize the threshold energy density, ε*TH*, to put as much of the available laser energy as possible into the ablation process, and to maximize the amount of energy leaving the ablation site within the ejected plume, which limits the residual energy left behind [9]. Residual heating below the ablation threshold and propagation of thermal and acoustic transients to the surrounding tissue make this a challenge [9]. In the current study, we analyzed tissue heating during ablation using a thermal camera and found no heating beyond physiological ranges. However, these data should be interpreted with caution as the frequency of the thermal camera could be too slow to detect fast temperature changes due to laser ablation. On the other hand, histology revealed thermal effects on maximum the first layer of cells adjacent to the PIRL incision with similar appearance in all samples, independent of ablation time and maximum temperature.

The most abundant vibrational chromophores in the human body are water (λ = 2.9 μm for the stretching and λ = 6.1 μm for the bending mode), collagen (λ = 6.1 μm for the amide I band and λ = 6.4 μm for the amide II band), and hydroxyapatite (phosphate modes around λ = 9.5 μm and also λ = 2.9 μm for the O-H stretch). Water is by far the most abundant component in tissues and, as mentioned above, displays the fastest ultrafast vibrational relaxation dynamics. In addition, water possesses the lowest critical temperature among the tissue matrix components so it can act as the most efficient ablation propellant [9]. For “matrix-continuous” soft tissues, such as dermis, cartilage or cornea, collagen fibrils are the main mesoscopic structural elements [15]. Their diameter is on the order of 100 nm and they are embedded within an amorphous matrix, the ground substance, which composed of water and proteinaceous components. Almost all the water in matrix-continuous tissues is located in the ground substance. The cornea is a typical “matrix-continuous” soft tissue [15]. The tissue optics in healthy and pathological cornea are dominated by Rayleigh scattering processes caused by the interaction of the incoming electromagnetic wave with the spatial distribution of the collagen fibrils [8, 12, 23]. For a wavelength of 1 μm, the total transmission is about 90%; however, the direct transmission is only about 50%. About 40% of the incoming light is scattered [12]. This is true for all existing FS laser platforms. These measurements show the potential benefits of shifting the wavelength towards the infrared when working in edematous cornea (and sclera).

At wavelengths around 1 μm typically used by clinical FS laser systems, a considerable fraction of the surgical laser beam is randomly deviated by optical scattering, and the beam quality of a surgical laser is thus strongly degraded [12]. Therefore, frequencies in the mid- and far-infrared ranges may overcome this limitation of current laser systems. Plamann et al.[12] have shown that, while the beam propagation in the cornea in the visible and near-infrared spectrum is dominated by optical scattering, within the optical window between 1.6 and 1.8 μm scattering was strongly reduced, and residual optical absorption dominated. However, the cutting was performed after applanating the cornea with a microscope coverslip to obtain a flat surface.

Although no previous data are available on the behavior of laser systems with emitting wavelength beyond 2.6 μm with regards to the relative contributions of absorption and optical scattering in corneal tissue, our current experimental set-up (3 μm wavelength) enables the minimization of optical scattering while permitting very good-quality incisions deep into the corneal stroma (Fig. 3A-B).

The plateau in ablation depth obtained with longer ablation time could be explained by diffraction properties of the laser beam with increasing tissue depth or it could be caused by effects of descemet’s membrane, i.e. acted as a barrier to the laser beam because of reduced water content in comparison to corneal stroma.

Another benefit of the wavelength shift to the mid-infrared range is the fact that wavelength beyond the water absorption peak centered at 1450 nm are inherently safe for the eye, inasmuch as only a negligible amount of the incident light reaches the retina [7].

In the mid-infrared range (wavelength λ = 3 μm), as our experiments reveal, DIVE-based PIRL technology permits deep cornea-penetrating cuts and, therefore, does not present any limitation to corneal transplant surgery.

Additionally, to the best of our knowledge, besides the non-mechanical excimer trephination using an erodible mask, the PIRL is the only laser system enabling contact-free penetrating cuts in corneal tissue [24–28].

Although optical scattering is stronger in sclera, similar arguments apply, and experiments are in progress to test the feasibility of PIRL for cutting sclera and for glaucoma surgery.

Here we report on the highly efficient ablation of healthy cornea using a 300-ps laser pulses tuned to the O-H stretch vibrational band of water molecules—a main component of corneal tissue. PIRL technology is still in its early stage. To date there are only two prototypes available. Despite these promising results, there are limitations concerning cutting features and applicability. The current PIRL output beam must be focused to a tight spot in order to achieve a significant photon density for ablation to occur; however, the prototype does not possess any autofocus function [16]. Hence, suboptimal focusing may occur, leading to decreased ablation efficiency and subsequent deposition of unfocused laser energy as heat.

To the best of our knowledge, this is the first report showing that wavelengths in the mid-infrared range centered at 3 μm are efficient for obtaining applanation-free deep cuts on healthy postmortem cornea. Since the fundamental action of the PIRL laser aims at O-H bonds, edematous (H_2_0 enriched) corneae should be ideal candidates for PIRL cutting. Therefore, we have initiated experiments focusing on cutting pathological tissues.

## Conclusion

Mid-infrared pulses are an effective tool for corneal incisional surgery, such as cornea transplantation. The pulses are tuned to one of the dominant vibrational states of the tissue (λ = 2.96 μm) with pulse durations that are sufficiently short (300 ps) to deposit heat through ultrafast vibrational relaxation but with intensities small enough to avoid plasma formation. Future wound healing and in vivo experiments will be needed to confirm this promising approach.
